# Evaluation of Access Disparities to Biologic Disease-Modifying Antirheumatic Drugs in Rural and Urban Communities

**DOI:** 10.7759/cureus.26448

**Published:** 2022-06-30

**Authors:** Nicholas J Peterman, Aksal Vashi, Devan Govan, Amrit Bhatia, Tejal Vashi, Brad Kaptur, Eunhae G Yeo, Alison Gizinski

**Affiliations:** 1 Medicine, Carle Foundation Hospital, Urbana, USA; 2 Medicine, Carle Illinois College of Medicine, Urbana, USA; 3 Medicine, University of Cincinnati College of Medicine, Cincinnati, USA; 4 Statistics, Social & Scientific Systems, Atlanta, USA; 5 Rheumatology, Emory University Hospital Midtown, Atlanta, USA

**Keywords:** rural vs metropolitan, healthcare inequality, medicare data, disease-modifying antirheumatic drugs, geospatial analysis

## Abstract

The American College of Rheumatology guidelines provides a strong recommendation for the use of biologic disease-modifying antirheumatic drugs (bDMARDs) when conventional rheumatoid arthritis treatments fail to meet treatment targets. Although bDMARDs are an effective and important treatment component, access inequalities remain a challenge in many communities worldwide. The purpose of this analysis is to assess nationwide trends in bDMARD access in the United States, with a specific focus on rural and urban access gaps. This study combined multiple county-level databases to assess bDMARD prescriptions from 2015 to 2019. Using geospatial analysis and the Moran’s I statistic, counties were classified according to prescription levels to assess for hotspots and coldspots. Analysis of variance (ANOVA) was used to compare significant counties across 49 socioeconomic variables of interest. The analysis identified statistically significant hotspot and coldspot prescription clusters within the United States. Coldspot (Low-Low) clusters with low access to bDMARDs are located predominantly in the rural west North Central region, extending down to Oklahoma and Arkansas. Hotspot (High-High) clusters are seen in urban and metro areas of Wisconsin, Minnesota, Pennsylvania, North Carolina, Georgia, Oregon, and the southern tip of Texas. Comparing coldspot to hotspot areas of bDMARD access revealed that the Medicare populations were older, more rural, less educated, less impoverished, and less likely to get their bDMARDs from a rheumatologist.

## Introduction

The 2015 American College of Rheumatology (ACR) guidelines for the treatment of rheumatoid arthritis (RA) provide a strong recommendation for the use of biologic disease-modifying antirheumatic drugs (bDMARDs) in combination with conventional disease-modifying antirheumatic drugs (cDMARDs) for patients who fail to meet treatment targets with cDMARDs alone [1]. Biologic disease-modifying antirheumatic drugs can also be used in the treatment of other disorders including connective tissue diseases such as systemic sclerosis (SSc), systemic lupus erythematosus (SLE), and Sjogren syndrome (SS), as well as in the treatment of inflammatory myositis, vasculitis, uveitis, and inflammatory bowel disease [2-4]. The addition of bDMARDs is efficacious in both slowing disease progression and minimizing disease activity [5-7].

Although bDMARDs are an effective and important component of common pathologies such as RA, inequity of bDMARDs access remains a challenge in many communities worldwide [8]. Previous studies have compared bDMARD usage across European countries and found a negative relationship between access to bDMARDs (influenced by many factors, including socioeconomic status, affordability, and valid prescription and reimbursement rules) and disease activity in patients with RA [9]. Communities facing disproportionately decreased access to bDMARDs could face worse disease activity in inflammatory arthritides and ultimately face worse long-term outcomes.

Rural American communities face several challenges in accessing healthcare from reduced facilities due to hospital closures, lower rates of insured status compared to urban areas, and a shortage of both primary care and specialty care physicians [10-12]. Previous works have assessed the impact of both the rural character of an area and DMARD use for various demographics in the United States (US) [13-16]. Looking abroad, urban communities in both Canada and Romania have significantly higher rates of bDMARD use compared to their respective rural counterparts [17,18]. If a similar relationship exists in the United States, it would be important to recognize and develop initiatives to address such a gap. Additionally, previous studies have determined outcomes of patients with rheumatic disease can be positively influenced by being treated by a rheumatologist compared to a non-rheumatologist, and patients are expected to have worse outcomes when their care is delayed [19,20]. Furthermore, the majority of rheumatologists are in metropolitan areas, while the majority of rural areas do not have a rheumatologist in their community [21]. Currently, there is no clear consensus regarding US national variations in bDMARD access and whether such variations are the result of fewer prescribing physicians available or physicians in underserved communities prescribing fewer bDMARDs.

In this study, we aim to conduct a retrospective nationwide analysis of Medicare bDMARD prescription rates and elucidate potential gaps in care. While previous studies have examined racial, economic, and other demographic variations in bDMARD prescription rates for local communities, there is insufficient literature examining national patterns of geographic distribution and community access to bDMARDs [22,23]. If all Medicare bDMARD prescriptions are geospatially organized, we expect: (1) a strong urban bias indicating significantly reduced access in metro and rural communities compared to city centers (urban, metro, and rural defined by the United States Department of Agriculture (USDA) rural-urban continuum (RUC) designations), and (2) variations in bDMARD prescription rates across the US to be attributable to fewer prescribing physicians servicing low access communities as opposed to lower quality care or physicians not prescribing sufficient bDMARDs. Categorization of locations with varying levels of bDMARD access will allow for further socioeconomic and demographic stratification of bDMARD access disparities in the US.

## Materials and methods

To provide a more robust analysis of nationwide trends in bDMARD access, this study combined multiple public access, county-level databases, including: Medicare Part D prescriber, US Census, Centers for Medicare & Medicaid Services (CMS) Medicare demographics, CMS chronic disease, and the USDA Economic Research Service’s (USDA ERS) databases [24-27]. Because these databases were deidentified and public access sources, no IRB was required. All datasets were obtained for the latest available years which were 2015-2019, with the exception of USDA urbanization metrics which were from 2013. Python (Python Software Foundation, Wilmington, Delaware, USA) was used to merge these databases resulting in a singular data frame organized by county with a total of 49 socioeconomic and bDMARD prescription-related variables. Of the 3109 counties in the contiguous US, 3072 were utilized in the analysis after filtering out counties that had incomplete data in any of the 49 variables of interest.

To properly compare and sum prescription patterns of the different bDMARDs, it would be improper to directly compare the total number of doses as different medications have different strengths and dosing regimens. Therefore, the total days of coverage prescribed (a base metric recorded in the CMS prescriber dataset) was used as the core metric for tracking bDMARD prescription patterns as it is standardized across all medications. All prescribing variables were scaled to the Medicare Part D populations of their respective county. The bDMARDs rituximab, abatacept, adalimumab, etanercept, infliximab, certolizumab, tocilizumab, and golimumab were tracked both individually and as a whole. The average yearly cost of bDMARDS per county per 10,000 Medicare Part D members was also determined. However, it does not represent the cost to the patient but rather the net cost to Medicare, including ingredient, dispensing, and sales costs. The specialty of the prescriber, the number of different prescribers, and the relative amount of bDMARDS prescribed by each specialty were also calculated for each county as core metrics in understanding who is controlling the bDMARD supply.

Centers for Medicare & Medicaid Services' demographic information, including average race, age, and gender breakdowns for each county from 2015-2019, was added along with CMS chronic disease data. Chronic disease data included the prevalence of various chronic diseases and risk factors in the Medicare community including obesity, tobacco use, diabetes, and chronic kidney disease, among others. The USDA ERS 2013 data on the latest RUC designations (a rank from 1 to 9) were used to identify each county on a spectrum from rural (rank > 3) to metro (rank < 3) to urban (rank = 1).

The completed dataset was then exported to GeoDa, a geospatial analysis program from the University of Chicago Champaign, to perform network analysis. Each of the 3072 counties was treated as a node in a network with their connections weighted based on the geospatial distance between them. Moran’s I statistic, with a null hypothesis that the bDMARD distributions would be spatially random, was used to identify statistically significant (p < 0.05) clustering across average yearly prescribed days of all bDMARDs per 10,000 Medicare Part D members and across each individual bDMARD. The Moran’s I statistic, specifically the local Moran’s I statistic that was utilized in this analysis, identifies and groups nodes (i.e., counties) into five potential categories: Not Significant, High-High, Low-Low, Low-High, and High-Low. Each designation (with the exception of Not Significant) is a combination of two attributes: the first represents a county’s value compared to the average county, and the second represents the average of a county's neighbors compared to the average county. Both values must be statistically significantly (p < 0.05) higher or lower than the overall average for a county to not be placed in the Not Significant group. For example, a High-High designation implies that both the county and the surrounding counties have a significantly higher than national average rate of bDMARD prescription days per 10,000 Medicare Part D members. If both attributes are matching (i.e., High-High or Low-Low) then these counties are known as spatial clusters and represent hotspots and coldspots, respectively, for the value of interest. If the attributes are not matching (i.e., Low-High or High-Low) these represent spatial outlier counties, which denote counties that are significantly different from their neighbors. These can represent transition zones and often demarcate shifts from rural to urban counties. The Moran’s I statistic for the average yearly cost of bDMARDS per county per 10,000 Medicare Part D members was used to group all counties into their statistically significant designations which were then exported back to Python where a one-way ANOVA was performed. This ANOVA compared the four groups across the 49 socioeconomic and bDMARD prescription variables to determine what demographics and communities were under- and over-served in access to bDMARDs. Statistical significance in this analysis was defined as p < 0.001.

## Results

**Figure 1:**
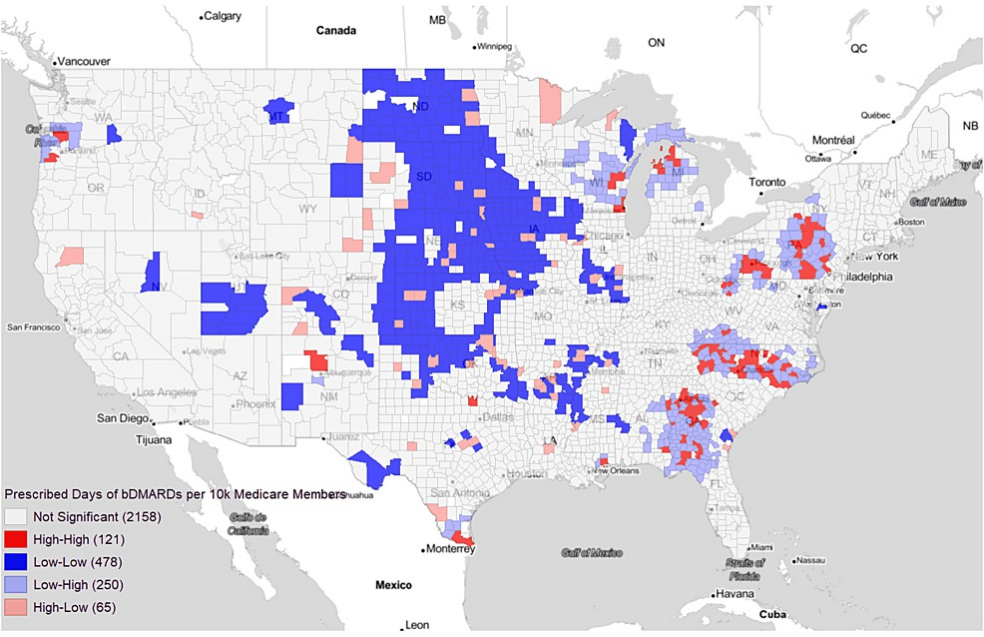
Prescribed days of bDMARDs per 10,000 Medicare members Visual representation of counties with Moran's I spatial outlier categories (p < 0.05) for average yearly prescribed days of bDMARDs per 10,000 Medicare Part D members. Gray areas signal non-significance in the geographic designation. bDMARDs: Biologic disease-modifying antirheumatic drugs

**Figure 2:**
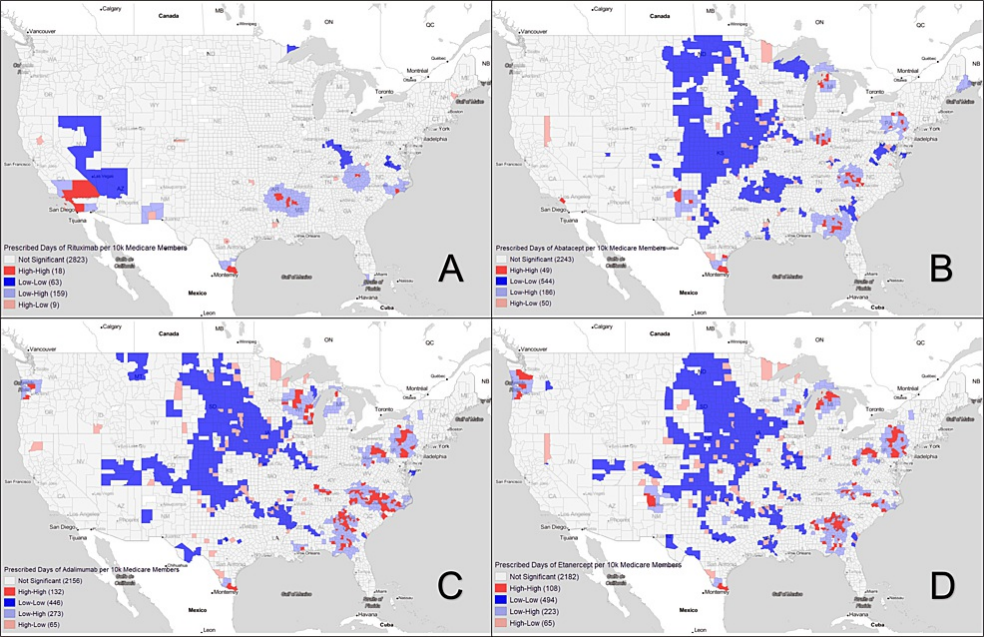
Prescribed days of individual bDMARDs per 10,000 Medicare members Visual representation of counties with Moran's I spatial outlier categories (p < 0.05) for individual bDMARDs — A: Rituximab, B: Abatacept, C: Adalimumab, D: Etanercept. Gray areas signal non-significance in the geographic designation. bDMARDs: Biologic disease-modifying antirheumatic drugs

**Figure 3:**
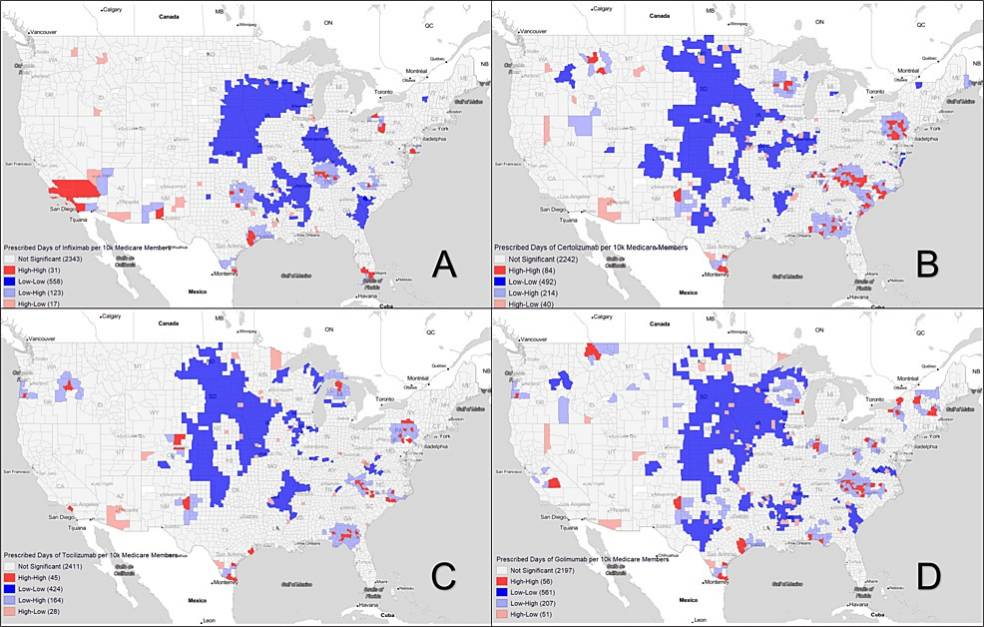
Prescribed days of individual bDMARDs per 10,000 Medicare members Visual representation of counties with Moran's I spatial outlier categories (p < 0.05) for individual bDMARDs — A: Infliximab, B: Certolizumab, C: Tocilizumab, D: Golimumab. Gray areas signal non-significance in the geographic designation. bDMARDs: Biologic disease-modifying antirheumatic drugs

Using Moran’s I for analysis of average county-level bDMARD prescription patterns from 2015 to 2019, we were able to reject the null hypothesis of spatial randomness and found clear disparities in access. Looking first to Figure 1, which is a Moran’s I plot of all bDMARDs, coldspot (Low-Low) clusters with low access to bDMARDs are located predominantly in the rural west North Central region, extending down to Oklahoma and Arkansas (804 ± 1607.39 days of bDMARDs per 10k Medicare members). Hotspots (High-High clusters) are seen in urban and metro areas of Wisconsin, Minnesota, Pennsylvania, North Carolina, Georgia, Oregon, and the southern tip of Texas (29,969.84 ± 26,648.3 days of bDMARDs per 10k Medicare members). Spatial outliers (High-Low and Low-High counties) were in the cities and large towns of primarily rural Midwestern states (21,007.46 ± 19,226.54 days of bDMARDs per 10k Medicare members) and in the urban and hotspot adjacent suburbs (1,308.76 ± 1,740.39 days of bDMARDs per 10k Medicare members), respectively. When prescription patterns were further broken down into individual bDMARDS (Figure 2 and Figure 3), these general clusters and outliers held true with the exception of rituximab, which avoided the Midwest altogether. Instead, it demonstrated hotspots in California, Arkansas, southern Texas, and North Carolina, and coldspots in Nevada, Arizona, and Kentucky.

**Figure 4:**
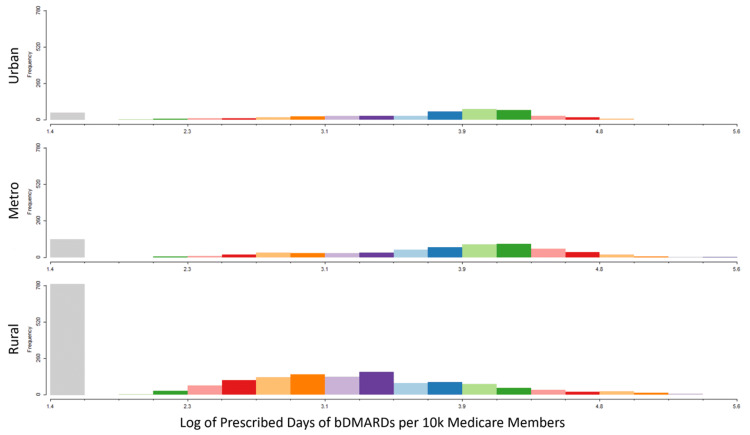
Log of prescribed days of bDMARDs per 10,000 Medicare members Paired histograms of the number of counties in (A) urban, (B) metro, and (C) rural areas. Gray bars indicate the number of counties in each classification with zero bDMARD prescriptions. bDMARDs: Biologic disease-modifying antirheumatic drugs

**Figure 5:**
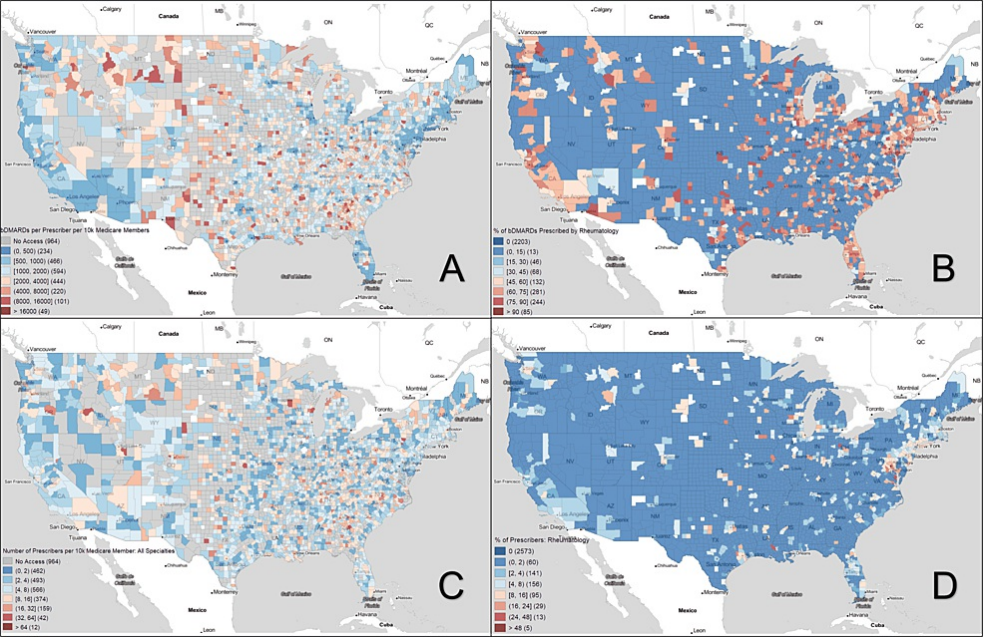
Geospatial analysis of bDMARD prescriptions in the United States (2015 to 2019) A: Number of days of bDMARDs prescribed per provider per 10,000 Medicare members per year on average. B: Percentage of bDMARDs prescribed by rheumatologists as a percentage of total bDMARD prescriptions. C: Number of prescribers of bDMARDs per 10,000 Medicare members for all specialties. D: Percentage of bDMARD prescribers who are rheumatologists. bDMARDs: Biologic disease-modifying antirheumatic drugs

A paired histogram of the scaled-to-population bDMARD distributions in Figure 4 was created to highlight the direct disparity between rural, metro, and urban areas. The distribution showed almost six times more rural counties had no access to bDMARDS compared to metro counties, which themselves had twice as many counties without access as urban counties. The left shift in the rural histogram indicates that these counties when they do have access are also proportionally receiving fewer bDMARDs compared to urban and metro counties, which appeared to have similar distributions in counties with access. Patterns in prescribers were explored in Figure 5 with key trends showing a larger proportion of bDMARDs prescribed by rheumatologists in areas previously designated as bDMARD hotspots.

The ANOVA displayed in Table 1 identified statistically significant differences in bDMARD prescription clusters across 46 of the 49 variables with a significance level of p < 0.001. Comparing coldspot to hotspot areas of bDMARD access, the Medicare populations were older (72.91 to 70.92 years old), more white (88.83% to 82.17% white), more rural (6.76 to 2.94 RUC), less educated (21.34% to 26.51% with college degrees), less impoverished (13.69% to 15.3% in poverty), and less likely to get their bDMARDs from a rheumatologist (2.57% to 53.12%). Interestingly, there was no statistically significant difference in the percentage of Hispanics, average per capita cost to Medicare, or the percentage of the uninsured (in the non-Medicare population) between hotspots and coldspots.

**Table 1:** ANOVA analysis of prescribed days of bDMARDs per 10,000 Medicare members ANOVA across all 49 socioeconomic and prescribing variables between Moran’s I determined county clusters (High-High, Low-Low, Low-High, and High-Low). bDMARDs: Biologic disease-modifying antirheumatic drugs, ANOVA: Analysis of variance, COPD: Chronic obstructive pulmonary disease, GED: General education development, SD: Standard of deviation

ANOVA Analysis of Prescribed Days of bDMARDs per 10k Medicare Members									
Cluster	High-High		Low-Low		Low-High		High-Low		P-value
Counties per Cluster	121		478		250		65		
Demographic Variable	Mean	SD	Mean	SD	Mean	SD	Mean	SD	
Average Medicare Age	70.92	1.26	72.91	1.67	70.35	1.27	71.79	1.61	2.38E-89
% Male	45.66	1.66	46.03	1.88	46.96	2.01	45.51	1.78	3.07E-13
% White	82.17	13.98	88.83	8.85	83.59	11.24	84.56	12.49	2.86E-13
% Black	11.94	11.64	4.3	7.78	11.74	10.49	7.1	7.65	3.55E-28
% Hispanic	2.83	9.07	2.69	4.03	1.98	4.37	4.4	10.91	0.022645
% Other Race	3.06	1.96	4.19	3.06	2.69	1.56	3.94	2.92	6.22E-13
Medicare Population Density	27.94	34.16	3.42	6.41	9.11	13.03	15.51	24.01	1.15E-44
Total Population Density	407.15	571.64	30.95	82.94	100.44	181.77	196.61	345.74	1.14E-42
Average Medicare Cost per 10k Members	95101889	9580310	97318951	15004047	95902837	11509615	96005637	13554571	0.29842
Metro (binary value 0,1)	0.76	0.43	0.14	0.35	0.31	0.46	0.63	0.49	5.74E-51
Urban (binary value 0,1)	0.25	0.43	0.03	0.16	0.09	0.28	0.05	0.21	3.83E-16
% Poverty	15.3	5.55	13.69	5.97	18.58	6.59	14.42	5.04	3.25E-22
Median Household Income	53252.58	12743.5	51564.96	9554.48	44954.49	9885.32	52809.79	9183.56	8.72E-19
Unemployment	4.9	0.95	3.62	1.38	5.59	1.45	3.78	1.02	5.61E-71
Uninsured	12.06	4.89	11.16	4.86	12.27	4.48	11.92	5.54	0.017009
Rural-Urban Continuum Code (integer 1-9, 1=most urban, metro<=3)	2.94	1.95	6.76	2.3	5.16	2.49	4.29	2.69	2.83E-56
% Without GED	12.45	5.46	10.77	5.41	15.96	6.48	10.93	5.8	1.05E-27
% With Only GED	31.73	7.77	34.25	5.72	37.6	6.07	30.84	6.45	1.54E-22
% Some College	29.32	4.34	33.64	4.71	29.34	4.87	32.95	4.99	8.67E-35
% College Degree	26.51	11.24	21.34	6.31	17.09	6.4	25.28	9.13	1.17E-31
% Medicare Alcohol Abuse	2.17	0.47	1.23	0.75	2.2	0.57	1.72	0.84	2.41E-74
% Medicare Asthma	4.9	0.79	3.45	1.17	4.61	0.98	4.09	1.28	4.05E-53
% Medicare Chronic Kidney Disease	23.92	4.02	19.6	3.44	24.07	3.73	20.5	3.64	9.52E-59
% Medicare COPD	12.27	2.82	11.65	2.78	14.22	3.36	11.75	2.48	1.95E-26
% Medicare Depression	18.89	2.3	15.85	3.06	18.39	2.81	17.93	3.5	2.22E-36
% Medicare Diabetes	27.8	4.5	24.63	3.98	29.61	4.2	25.03	4.32	3.53E-49
% Medicare Drug Abuse	3.26	1.16	1.58	1.25	3.35	1.61	2.45	1.44	3.27E-63
% Medicare Stroke	3.77	0.63	2.73	0.87	3.71	0.88	3.06	0.98	2.69E-54
% Medicare Osteoporosis	6.18	1.46	5.45	1.83	5.46	1.55	6.21	2.13	8.41E-06
% Medicare Tobacco Use	10.41	2.81	8.11	2.61	11.85	2.81	9.23	2.38	6.75E-62
% Medicare Chronic Pain/Fibromyalgia	19.22	2.71	18.9	3.72	20.25	3.35	19.68	2.93	1.26E-05
% Medicare Migraine	3.12	0.6	2.3	0.76	3.02	0.71	2.73	0.82	1.69E-42
% Medicare Obesity	19.86	4.97	13.42	4.07	20.72	5.52	14.69	4.08	5.37E-85
Prescribed Days of bDMARDs per 10k Medicare Members	29969.84	26648.3	804	1607.39	1308.76	1740.39	21007.46	19226.54	6.85E-129
Cost of Part D Drugs per 10k Medicare Members	1.16E+08	76730936	28488930	32724257	40398613	37050660	1.08E+08	1.25E+08	2.62E-64
Prescribed Days of Adalimumab per 10k Medicare Members	14104.41	12109.45	450.8	1093.59	770.68	1154.73	11428.47	11219.53	3.14E-126
Prescribed Days of Etanercept per 10k Medicare Members	11551.29	12427.73	293.77	780.68	462.44	825.53	7300.38	8648.18	6.64E-95
Prescribed Days of Infliximab per 10k Medicare Members	165.62	688.7	6.41	79.3	12.35	98.83	38.69	160.04	7.90E-08
Prescribed Days of Certolizumab per 10k Medicare Members	898.57	1155.36	10.39	71.45	18.25	72.36	566.02	919.29	2.10E-69
Prescribed Days of Golimumab per 10k Medicare Members	432.77	720.82	3.67	31.04	12.69	77.22	291.31	564.62	4.53E-45
Prescribed Days of Abatacept per 10k Medicare Members	1773	3597.03	17.76	160.31	26.31	120.7	760.87	1404.84	3.42E-35
Prescribed Days of Rituximab per 10k Medicare Members	160.42	833.25	16.65	188.23	1.35	11.48	56.26	429.77	0.00026
Prescribed Days of Tocilizumab per 10k Medicare Members	883.75	1554.13	4.55	36.78	4.69	27.18	565.47	1084.77	7.48E-44
Cost of bDMARDs per 10k Medicare Members	4952699	4375436	134323.1	273511	229620.2	310165	3522057	3157312	3.05E-130
% of bDMARDs Prescribed by Rheumatology	53.12	31.53	2.57	13.23	4.91	17.78	46.23	35.79	1.54E-128
Number of Prescribers per 10k Medicare Member: All Specialties	14.88	11.04	1.69	3.36	2.41	3.2	17.9	22.06	4.73E-86
Number of Prescribers per 10k Medicare Member: Rheumatology	0.14	0.32	0.01	0.1	0.03	0.12	0.32	0.83	3.81E-19
% of Prescribers: Rheumatology	1.08	2.27	0.36	2.89	0.77	3.61	1.95	3.51	0.000376
bDMARDs per Prescriber per 10k Medicare Members	4775.73	5760.03	1089.63	2500.34	1214.4	2154.53	6633.96	12467.15	5.50E-29

## Discussion

The majority of existing literature assessing bDMARD access in the US has focused on the geographic barriers facing patients with rheumatic diseases in rural communities [13-16]. Our study builds on these findings to a more precise county-level distinction. The degree to which a community is urbanized cannot be overstated in its influence on bDMARDs access, both as an influencing factor and potential confounding variable.

Much of the statistically significant socioeconomic differences between hotspots and coldspots of bDMARD access are derived from the vast socioeconomic differences between urban/metro and rural counties. Although these correlations may be obvious in describing the rural/urban continuum, they are still more accurate in describing the attributes of the communities most affected by bDMARD disparities. It is interesting to note, however, the presence of an unexpected urban/metropolitan divide, which can be seen with a bullseye-type pattern as seen above in Figure 1. It appears the high bDMARD urban city centers have a “pulling” type effect on prescription frequency, resulting in lower-than-average bDMARD rates in their immediately neighboring metro areas. We propose a few possible explanations: large hospitals containing more prescribers outcompeting surrounding areas, physicians billing Medicare from the hospital location rather than outpatient location, and suburban patients driving into a city for their physician but receiving the medication elsewhere. This final theory would also explain the geospatial High-Low outliers seen in the Midwest, where the few areas with bDMARD prescribers have to service a far larger region than just their community, effectively acting as healthcare hubs. These findings confirm and extend upon those of the American College of Rheumatology’s 2015 Rheumatology Workforce Study identifying severe shortages of rheumatologists across the country with higher rheumatologist-to-population ratios in urban/metropolitan areas, while rural/"micropolitan" (less than 50,000 people) populations often depend on rheumatologists over 100 miles away [28]. This data clearly shows that the lack of rheumatologists and shortages in certain parts of the country are leading to a disproportionate or complete lack of access to appropriate therapy.

Studies suggest that worldwide, the socioeconomic status of a community and the direct affordability of the medications for patients are the biggest predictors of bDMARD access [9]. Direct patient cost is often hypothesized as a core prohibitive factor in access to bDMARDs for various communities in the US. By the nature of our dataset only covering prescribed medications that were covered by Medicare, this normally influential factor has been controlled in our analysis. This study demonstrates that cost may play a lesser role in access to bDMARDs than traditionally thought: hotspot areas of bDMARD prescription rates were more impoverished than coldspot areas (as evidenced above in Table 1). Although, it is important to note that this is true as a nationally averaged county-level aggregate. While bDMARDs are highly expensive to Medicare, the lack of a statistically significant difference in overall per capita Medicare spending when comparing bDMARD hotspots and coldspots either suggests that either (1) bDMARD expenses are proportionally minuscule compared to other healthcare expenses at a county level, or (2) that communities with lower rates of bDMARD prescriptions/expenses billed to Medicare are perhaps offsetting the difference in cost by, in turn, incurring additional costs related to their disease progression.

The core driving force for the geographic disparities seen across our analysis is not the interplay of poverty and the high monetary cost of bDMARDs but instead the access to a rheumatologist. While rheumatologists only accounted for 1.08% and 0.36% of all specialists who prescribed bDMARDs in hotspot and coldspot areas, respectively, when comparing the percent of bDMARDs that are prescribed by rheumatologists, the proportions are 53.12% and 2.57%. Our interpretation of this finding is that in both hotspot and coldspotmany non-rheumatologists willho will occasionally prescribe the initial dose of medication to patients. But the difference between the high- and low-access areas is that the high-access areas have rheumatologists that manage the majority of patients’ long-term bDMARD care while low-access areas do not. Thus, one explanation for the overall disparity is a lack of access to specialized physicians that are comfortable handling long-term prescriptions of bDMARDS.

There are several limitations to the analysis conducted in this study that is important to acknowledge. First, the core of the data is derived from Medicare records, and while they can serve as estimators of trends in the overall population, the Medicare population is not a proportional random sample of the US population. Analysis of Medicare patients does, however, provide valuable information in guiding future policy as this population makes up a substantial portion of federal healthcare spending [29]. Second, while this data is averaged across five years to remove outliers and sampled from the latest available national datasets, it is from 2019 and may no longer accurately reflect the ever-growing medical infrastructure of the country in its current state. Third, while the county-level analysis lends itself well to macro-level national trends such as the clear urban/rural divide, there is likely masking of disparities in impoverished inner-city areas that should not be ignored. Since inner-city areas would naturally be grouped in the same county as more wealthy areas in most cities, potential lack of access would be averaged out and not seen in this analysis. For example, previous literature assessed that amongst Social Security Disability Insurance beneficiaries, bDMARD use was lower among Black Americans than white Americans [30]. Given the county-level grouping utilized by this study, Black Americans were identified to have significantly higher populations in hotspots versus coldspots. Thus, although our data show it is predominately white and that rural areas need the greatest outreach, we cannot discount pockets of disparities in high minority areas of cities that were outside the scope of our data. Finally, because this analysis tracks community trends on a national level and does not track individual patients and their health outcomes, the statistically significant conclusions will show correlations but we cannot make conclusions about causality.

## Conclusions

Despite its limitations, this study provides a comprehensive county-level statistical geospatial analysis of bDMARD access across the United States. The same study may be replicated using updated data released by the Centers for Medicare & Medicaid Services (CMS) as it becomes available. It will also be of interest to determine whether these inequities in geospatial access across the US are improving or worsening with time if more years of analysis are included. This insight into yet another health disparity facing rural communities and the descriptive mapping of relative bDMARD drug inequities can be used to guide further research such as creating initiatives by the CMS and the US Department of Health and Human Services to promote rural rheumatologic practice and improve access to care in rural areas of the US.
